# The importance of power, context and agency in improving patient experience through a patient and family centred care approach

**DOI:** 10.1186/s12961-019-0487-1

**Published:** 2020-01-23

**Authors:** Josephine Ocloo, Joanna Goodrich, Hiro Tanaka, Julia Birchall-Searle, Derek Dawson, Michelle Farr

**Affiliations:** 1 Centre for Implementation Science, Health Services, Population and Research Department, Institute of Psychiatry, Psychology & Neuroscience (IoPPN), London, UK; 2 National Institute for Health Research (NIHR) Applied Research Collaboration South London (NIHR ARC South London) at King’s College Hospital NHS Foundation Trust, London, UK; 3 The Point of Care Foundation, 3rd Floor, CAN Mezzanine, 7-14 Great Dover Street, London, SE1 4RY UK; 4 Aneurin Bevan University Health Board, Royal Gwent Hospital, Cardiff Road, Newport, NP20 2UB UK; 5 Manchester University NHS Foundation Trust, Manchester, UK; 6 Stoke-on-Trent, UK; 7 The National Institute for Health Research (NIHR) Applied Research Collaboration West (NIHR ARC West) at University Hospitals Bristol NHS Foundation Trust, Bristol, UK; 8 Population Health Sciences, Bristol Medical School, University of Bristol, Bristol, UK

## Abstract

**Background:**

Research shows that the way that healthcare staff experience their job impacts on their individual performance, patient experience and outcomes as well as on the performance of organisations. This article builds on this literature by investigating, with multi-disciplinary clinical teams as well as patients and relatives, what factors help or hinder changes designed to improve patient experience.

**Methods:**

Qualitative research looking at patient- and family-centred care (PFCC) on two care pathways (stroke and hip fracture) was conducted in England and Wales. A realist approach combined with participatory action research was used to account for the complexity of organisational context and power relations. Multiple methods were used, including documentary analysis, participatory steering groups with staff and patient representatives, observations of the care pathways (*n* = 7), staff and patient and relative focus groups (*n* = 8), and hospital staff, patient and PFCC staff interviews (*n* = 47).

**Results:**

Findings highlight multiple factors that support and hinder good patient experiences. Within individual care, paternalistic values and a lack of shared decision-making and patient-centred care still exist. Supportive interdisciplinary teamwork is needed to address issues of hierarchy, power and authority amongst staff and managers. At the organisational level, key issues of waiting times, patient flow, organisational resources and timely discharge affect staff’s time and capacity to deliver care. In addition, macro contextual factors, such as finance, policy, targets and measures, set particular limits for improvement projects.

**Conclusions:**

Given this context, improving patient experience needs to go well beyond small-scale projects at the micro and meso level to incorporate a more critical understanding of systems, the wider organisational context and how power operates at multiple levels to enable and constrain action. In order to more meaningfully understand and address the factors that can help or hinder activities to improve patient experiences, PFCC frameworks and methods need to account for how power inequities operate and require the adoption of more participatory co-produced and empowering approaches to involve patients, relatives, carers and staff in improving complex healthcare environments.

## Introduction

Staff and patient experiences are inextricably intertwined in delivering good patient and family-centred care (PFCC). Staff experiences can impact on their individual performance, patient experience and outcomes, and the performance of organisations [1–4]. Many international studies support the links between staff wellbeing and engagement and patient experience and satisfaction [5–9]. Good quality work environments have been found to address health inequities with patients and staff [10]. This is particularly important for some staff, as inequities for groups such as Black, Asian and minority ethnic (BAME) or disabled staff can be considerable [11, 12], as they can be for patients [13, 14].

### Patient experience

This evidence on staff experience sits beside longstanding strategic priorities to improve patient experience and to place patients at the centre of healthcare and decision-making [15]. Patient experience incorporates both the relational and functional aspects of care (the former regarding the two-way relationship between caregiver and patient and the latter regarding what is done to the patient) [16]. In the United Kingdom, this approach has been used to understand and improve patient experiences [17]. Despite this context, whilst patient experience is one of the central pillars of quality in healthcare improvement [18], it appears to be the ‘poor relation’ [19] to the other two main quality components — clinical effectiveness and safety — and is not always regarded as equal [17]. Further studies are needed that focus on the most effective ways to improve patient experience [19]. Patient experience frameworks tend to be situated within a positivist-empiricist approach, with the monitoring of patient experience largely dominated by quantitative surveys. However, conducting patient surveys about service experiences does not necessarily lead to quality improvements [20, 21]. Qualitative approaches such as patient stories can capture the interest of staff, yet they have been ‘underexploited’ as a way to improve care [21].

Patient experience is intrinsically connected with the principles and practice of PFCC [22]. Core principles of PFCC are the empowerment and engagement of patients, families and healthcare providers throughout the healthcare system “*with each vital to the delivery of quality and safe care*” [23]. The PFCC methodology advocates improving patient experience by staff shadowing patients as a way to view “*every care experience through the eyes of the patient and family*” [24]. PFCC methodology draws upon quality improvement methods such as care flow (process) mapping, shadowing [25], involvement of different caregivers, and data gathering and measurement [24]. However, less emphasis is placed in the PFCC methodology on involving patients in improvement efforts or on how to improve services within uneven and inequitable healthcare hierarchies. PFCC, as an improvement method, tends to focus on improvements at the micro, individual and team level, whilst insufficiently looking at inequities in power impacting staff and patients and how policy and organisational contexts shape healthcare environments. This research article examines the PFCC methodology and practice as an improvement method that uses patient experiences to improve services and looks at what helps or hinders changes designed to improve patient experience, with a specific focus on power and context.

The theoretical approach of this article aligns with realist research in healthcare that highlights the importance of power relations [26] and understanding how structure may both constrain or enable individual agency [27]. It critically examines the PFCC methodology, particularly given the fact that considerable emphasis has been placed on active meaningful and partnership approaches to involving patients and families across all western healthcare systems as central to improving patient experiences [4, 17, 19, 28]. However, current models of patient and public involvement (PPI) in NHS practice have been critiqued for being tokenistic, atheoretical and lacking in critiques about power, inclusivity and diversity [29, 30]. Whilst some writers have set out ways to develop more inclusive models of PPI [31, 32], in practice, evidence across clinical commissioning groups and NHS England shows that the dominant demographic in terms of PPI in the work of the NHS still tends to be white, middle class, educated and older people [33]. There is now increasing recognition at a policy level about the need for approaches to involvement that illustrate the importance of wider social drivers and inequalities that constrain healthcare and the way they exclude many of the groups with the poorest health outcomes from healthcare involvement processes [33].

Whilst PFCC literature suggests using complexity theory to account for social contexts [34], this has so far not addressed the need for a broader and more critical whole systems approach to understanding the power relationships that permeate all healthcare systems. Some of this overlooking of power may derive from the methodology from which the PFCC and patient experience methodology derives. Patient shadowing is rooted in ethnographic and interpretivist perspectives. However, by focussing closely on individual patient experiences, insufficient attention may be directed toward the structural and cultural dimensions within which these subjective experiences are shaped [35]. Further accounting of the social and organisational context is therefore needed, particularly of the hierarchies and unequal power dynamics that exist within healthcare [36].

### Realist social theory and power

The research study used realist social theory [37, 38] to take into account the complexity of the organisational context we were looking at, namely the social, policy and institutional structures and power relations that affect healthcare staff’s abilities to design and implement interventions to improve patient experiences [26]. Realist social theory [37–39] conceives of agency as people having an active and reflective nature, who can both habitually and purposefully act, depending on how they internally reflect on their social circumstances. Our unique identities enable us to be active agents that reflexively evaluate how we can act on our ‘personal concerns’ within particular social contexts [40]. Realist social theory enables an analysis of human action and its consequences, but it does not prioritise agency nor recognise contextual constraints. People’s actions can be constrained or enabled by their structural and cultural contexts, our actions are always situated within society. Agency is “*our gift to society … with society, through society and in society — but it can never be society’s gift to us*” ([39], p. 305). Social structures (material and social resources including roles, rules and systems) pre-exist and shape people’s actions; through people’s interactions they can then reproduce or change these social structures. People may have limited capacity for action within their specific social contexts, but their actions are not predetermined by structural and cultural contexts [37]. Instead, an analysis is conducted of how structure and agency intertwine to create stasis or change.

It has been highlighted that nursing research often emphasises agency and has neglected the structural issues in healthcare that can exert a powerful influence on how people behave [35]. Realist social theory conceptualises that both social structures and people have power [41]. Whilst power can be used to dominate others, it also has the potential to be a positive sum and can be used to create emancipatory and empowering social changes with different people benefitting from its use [42]. This more emancipatory use of power can be “*productive, transformative, authoritative and compatible with dignity*” [43]. A theoretical analysis of power needs to conceptualise both power as domination, and individual and collective empowerment, and how these power dynamics may interconnect [44]. Social interactions, the people involved and the structures within which social processes are set, all influence power dynamics and the extent to which agents may be empowered or dominated [41, 42, 45]. In healthcare, power, structure and agency shape both staff and patient experiences in different ways; whilst hospital hierarchical systems can be oppressive, they also create patient empowerment [46]. Patient empowerment can be an ongoing process, where active patient roles, information and knowledge, and positive, caring communications with professionals can all empower patients within health systems [47]. These patient experiences have their antecedents in staff well-being and positive working environments, where staff themselves are empowered to enable high quality patient care [3]. Yet, the most recent NHS Staff Survey shows that the number of staff who say that their organisation is taking positive action on staff wellbeing is falling [48]. The extent to which staff and patients can challenge and change institutions to facilitate patient-centred care can be a contingent process, dependent on institutional cultures and resources, networks, relationships, and staff’s ability to instigate change [26].

## Methodology

The findings presented here derive from qualitative research that took place between January 2013 and February 2014, conducted as part of a wider PFCC programme at the King’s Fund that covered 11 hospital sites. The programme was funded jointly by The King’s Fund and the Health Foundation. The PFCC Methodology and Practice consists of a six-stage process [49], initially exploring service experiences through empathetic understandings with the aim to then improve people's experiences of care. The six stages are as follows: (1) select a care pathway; (2) establish a guiding council; (3) understand the care pathway through patients’ eyes (using shadowing, care flow mapping surveys, reports and stories); (4) develop a ‘cross-functional and cross-hierarchical’ working group; (5) create a shared vision of the ideal experience; and (6) develop improvements and solutions [49].

This article focuses on two care pathways (stroke and hip fracture; see Appendix 1 for further background information on the composition of the care pathways studied and patients using them) that took part in the PFCC programme, at a Hospital Trust in England and a Health Board in Wales. Independent research looked at how teams at these sites actually implemented the PFCC method they were taught (at the King’s Fund PFCC learning events) and what helped or hindered staff’s abilities to improve patient experiences. The study methodology was not aimed at formally evaluating the PFCC approach used in the case study sites but was conducted as a way of understanding the deeper contextual factors impacting on changes to improve PFCC in two clinical areas at these sites. Ethical approval was sought and received from the National Research Ethics Service, London, Bloomsbury Committee, reference number 13/LO/0827. The research project was overseen at the King’s Fund level by a range of stakeholders connected to the project but separate to the management of the King’s Fund PFCC work.

The key research question was ‘What are the range of factors/processes within clinical microsystems (stroke/hip fracture) and at a wider context level that enable/impede staff teams to make changes/improve patient and family experiences of care (outcomes)?’ Within this, four sub-questions were considered, as follows:

1. What individual and team behaviours and attitudes result in improvements in patient and family experiences?
2. What systems/structures and processes enable/impede individuals and teams in improving patient experience?
3. What outcome measures enable agents to understand if changes have impacted patient and family experiences of care?
4. How do power dynamics and relational factors influence and impede change processes for staff/patients/families that are aimed at improving patient and family experiences of care?

### Participatory action research

We combined the above approach with participatory action research (PAR), which aims to challenge monopolies on the definition of knowledge [50] and create shifts “*in the balance of power in favour of poor and marginalized groups*” [51]. PAR enabled collaborative working within the case studies by informing the development of Research Advisory Groups (RAGs) and Focus Groups, the involvement of lay and professional members in conducting the research and in developing action workshops at the end of the research. Membership of RAGs included doctors, nurses, senior managers, service improvement specialists and patient representatives (two on the stroke pathway and one on the hip fracture pathway) as well as the researchers (JO, MF) on the pathways involved. The patient governor and stroke patient on the Stroke RAG was also involved in collecting data in the focus groups, conducting interviews and is an author on the paper. The RAGs guided the research and provided a space to plan and reflect on the data collection and the research process as well as to comment on findings by groups with very different perspectives. Everyone was also asked to keep a reflective log about their experiences in the group and people were free to share anything they thought was relevant at meetings.

Multiple qualitative methods were used, including analysis of key documents, e.g. key PFCC documents, key clinical guidance/targets on stroke/hip fracture care, and other performance data to measure patient experiences such as the Friends and Family Test in England [52]. Observations/shadowing of the care pathways (from accident and emergency (AE) to the ward) were completed to understand the patients’ journey and how staff provided patient care (*n* = 7). The observations and shadowing data were important in enabling researchers to understand the care pathways at both sites from AE to wards, through to discharge and the importance of multi-disciplinary teams in caring for patients. The shadowing process concentrated on pathway design (walking the route) and the observations focused on the experiences of five patients on different parts of the pathway from AE to the wards.

Three focus groups were held with staff (*n* = 16) and 23 staff interviews were conducted, including 2 noted conversations. Nineteen patient interviews were conducted (some relatives were present during interviews but were there as support for the patient) and 5 focus groups were held with 14 patients and 8 relatives (see Table 1 for full details of the methods used and data collected, Table 2 on different types of healthcare staff involved). Data collection ended once the preidentified numbers for interviews and focus groups were obtained and saturation levels were reached, with key research participant involvement identified. Focus group data were used to map responses from staff, patients and relatives onto key areas of the clinical pathways (such as pre-hospital and ambulance care, AE, the ward environment and post-hospital care) to understand different pathway experiences.

**Table 1:** Methods table

	Observations of 7 patients & pathway shadowing x 2	Number of staff participants in 3 focus groups	Number of patient participants in 5 focus groups	Number of relatives in 5 focus groups (relatives accompanying patients)	Number of staff interviews, including 2 noted conversations and PFCC staff interviews	Patient interviews	Research action group meetings
Total	7 observations, 2 pathways shadowed	16	14	8	28	19	8
Researchers involved	Patient observations 2 people (JO, DD) Pathway Shadowing x 2 3 people (JO, MF, DD)	3 (JG, JO, MF)	3 (JO, MF, DD)	3 (JO, MF, DD)	2 (JO, MF)	3 (JO, DD, MF)	3 (JO, DD, MF)
Length of time	Patient Observations took place over 5 different days Totalling 27 h (5/8/13, 9/8/13, 20–21/1/14) 22/1/14 Shadowing of pathways took place over 4 separate days (5/8/13, 9/8/13, 23/10/2013, 3/12/14) Totalling 20 h	1–2 h	2 h		Approx. 1 h	Approx. 1 h	2 h meetings

**Table 2:** Staff roles^a^

Staff role	Numbers
Consultant	4
Improvement Lead	1
Domestics	2
Ward Manager	1
Quality Nurse	1
Discharge Nurse	1
Discharge Facilitator	1
Nursing Assistants	2
Health Support Worker/Health Care Assistant/Ward Assistant	3
Sonographer/Radiographer	1
Technical Assistants	2
Physiotherapists	3
Occupational Therapists	2
Ward Sister	2
A&E Practice Educator	1
A&E Nurse	1
A&E Clinical Lead Nurse	1
Administrator	1
Matron	1
Surgeon	1
Deputy Sister	2
Staff Nurse	3
Advanced Nurse Practitioner	2
Total	39

^a^Specific roles are not identified separately by pathways in order to maintain the confidentiality and anonymity of the participants involved. Demographic data for staff, patients and relatives was not recorded systematically using a monitoring form and we have chosen not to identify staff by gender, again for confidentiality reasons

Health professionals contacted stroke and hip fracture patients from hospital databases to identify and invite potential focus group and interview participants, (with relatives invited if patient participants wanted this). The purposive sampling process aimed to include patients of different ages and genders, with an emphasis on those with ischaemic (blood clot) strokes rather than haemorrhagic strokes. With hip fracture patients, most participants were female, reflecting the demographic profile of these patients, with patients with dementia, in nursing homes or sheltered accommodation excluded from the selection process. We aimed to include a selection of patients with a range of experiences (good and bad) of care. However, we were only able to interview one patient who had made a complaint because of the lack of formal or documented complaints at the case study sites. We also only interviewed one person from a BAME background due to lack of information on databases and response to invitation letters. Patients who were interviewed were selected because they were unable to attend the focus groups and/or because they met some other aspect of the selection criteria. Staff participation was self-selecting, based upon emailing all staff on the two care pathways (e.g. doctors, nurses, healthcare assistants, occupational and physiotherapy staff, and selective domestic staff on wards) and then including all those who responded in focus groups or interviews. In addition to these two case studies, five interviews were conducted with senior members who were part of managing the PFCC Programme at the King’s Fund about the PFCC process and specific implementation issues at the two sites.

### Coding and analytic framework

A specific coding and analytic framework (Appendix 2) was informed by Archer’s [37] realist social theory and the PARiHS (Promoting Action on Research Implementation in Health Services) framework [53, 54]. We used elements of the PARiHS framework [53, 54] to situate Archer’s [37, 38] sociological theory within healthcare and to more finely analyse its different contextual elements.

Specific clinical contextual codes were developed using the stroke and hip fracture pathways to analyse patient experiences through the service (e.g. X-ray and theatre for hip fracture, specific treatments for stroke). Data from different sources was integrated at the analysis stage [55], using the two clinical pathways as a framework to understand both staff and patient experiences. This coding framework supported the realist method of retroduction to understand what individual and team behaviours and attitudes within what systems and structures resulted in improved patient experiences. The quality of the findings was ensured through use of Dedoose software to dual code interview and focus group transcripts with the involvement of a research team (made up of three researchers) who discussed any differences of opinion within analytic codes. All other data (e.g. key documents, observational and shadowing data and RAG minutes) were read through separately by the lead researcher to identify key themes and discussed with a second researcher. Drafts of key findings were then shared with the research action groups at the case study sites to strengthen validity and enable further refinement.

## Findings

A total of 33 patients were interviewed and participated in focus groups (8 relatives also participated in the study, 2 of whom were unequivocally happy with their care); 12 patients described being very happy with their care (4 on the stroke pathway and 8 on the hip fracture pathway) and 21 described a mixture of good and poor experiences of care (13 patients on the stroke pathway and 8 on the hip fracture pathway). Interview quotes are labelled according to the type of interviewee and numbered to illustrate the different people who are quoted. We discuss the first research question in relation to individual, patient-staff interactions and team dynamics that made a difference to how patients experienced their care. The second research question explores wider structural factors that limited staff’s ability to create changes to improve patient experiences. The third research question on outcome measures explores how staff gathered and used evidence and data about patient experiences. Through this analysis, we answer the fourth research question about the power dynamics that arose at different structural, cultural and agential levels in the case study Trusts and their impact upon staff as well as patients and carers, drawing these issues together in the Discussion section.

### What individual and team behaviours and attitudes result in improvements to patient and family experiences?

Patients and their relatives at both sites described good and poor experiences of care. Patients who had good experiences of care spoke of acts of kindness, compassion, respect, empathy, emotional support, caring and efficiency that made a key difference to their care.

“*They were actually waiting for us as the ambulance pulled up, I mean I’ve only seen that on Casualty and Holby City. We could not expect any better service at all*” (Stroke AE, relative 1).

“*On one occasion, a member of staff got down on her knees and washed and creamed* [the patient’s] *feet, which* [she thought] *was wonderful*” (Hip fracture ward, patient 2).

“*The cleaners, they’d run errands for some of the elderly ladies who never had visitors … they’d go off duty and next day they’d come in with fresh soap for them and something they like to smell, lavender and things*” (Hip fracture ward, patient 3)*.*

Patients also described positive experiences of communication where they and family members were “*informed*”, “*given information verbal and written and visited by senior members of the clinical team after surgical procedures*”.

One patient described how they found staff doing everything they could in the circumstances:

“*They did so many tests it was unbelievable, it was as if we’d got every specialist running around, there was no waiting for this, wait for that, everything was done*” (Stroke AE, patient 4).

Conversely, patients who experienced poor care (described as behaviour lacking in compassion, unkindness or rudeness, bullying, gossiping, not listening), said they felt considerably disempowered:

“*The only thing I did not like was them night nurses gossiping and they couldn't be bothered to answer that bell … She said ‘you don’t need a bedpan’. I said ‘I will do, by the time I get out now I’ll be wetting myself and I’ve waited long enough for you’. She said ‘we’re too busy’*” (Hip fracture ward, patient 6).

“*I tried to explain how I was feeling at this moment in time, and all he could do was complain about the NHS*” (Stroke outpatient, patient 7)

Overall, 21 out of 33 patients across both sites described feeling lonely and isolated in their care. They raised concerns about poor or inadequate communication or information by doctors and nurses that did not allow them to feel listened to, ask questions or to discuss their concerns, test results, clinical condition, care and treatment or the taking of new drugs:

“*In my opinion, you’re told what’s going to happen to you and you ain’t got no say in the matter*” (Hip fracture ward, patient 8)

“*Just being acknowledged that I existed would have been really very nice*” (Stroke ward, patient relative 9)

Policies that were supposed to support patient- and family-centred empowerment did not always translate into patient and relatives everyday experiences: A relative of a stroke patient who had experienced various difficulties in getting her concerns and then her formal complaint addressed and in trying to use services such as the Patient Advice and Liaison Service, said: “*Didn’t know which PALS* [Patient Advice and Liaison Service]*, so there was one for North-X and one for South-X and so I wrote to all of them and I got no joy from anybody*” (Stroke relative with concerns across the pathway, 10).

Communications with patient and family members could be particularly problematic:

“*He was suffering from dementia and he couldn’t communicate with anybody … he was so terribly lonely, he was crying … they* [nurses] *could have tried to communicate with him better than what they did … They were just going in with his medicine … saying, ‘Hi X’ and that was it, out again*” (Stroke ward, relative 11).

“*The worst thing was when they actually thought I was dying* [in intensive care]*, instead of waiting for my husband … My youngest daughter was eighteen. She was at the hospital all the time; she wanted to know what was going on. So she sat there and sat there … and my husband’s not there and family … And, instead of waiting, when the consultant came round, he told my eighteen-year-old, ‘Well, we don’t think your mum’s going to live’. And I think that’s a terrible thing to say to a young girl*.” (Hip Fracture, Intensive Care Unit patient about communication with her family, 12)

However, patients’ agency to have a say in their care could clearly be constrained by time pressures, on both wards:

“*Generally, she felt the nurses didn’t explain or give her info about her fracture properly, how big an operation it was. They just didn’t seem to have the time and said they would come back to her about this but didn’t*” (Hip fracture ward patient 13).

Additionally, after discharge in outpatient clinics, patients commented about the lack of opportunity to ask questions: “*There wasn’t the time, they were more interested in in and out*” (Stroke patient 12).The husband of a stroke patient noted, with respect to communicating with his wife while she was “*out of it all*” with her stroke, “*There was no backup at all* [for his wife]*, she seemed to be a nonentity*” (Stroke ward patient relative 14)*.*

However, staff’s own individual values and attitudes could shape the communication process with patients and their families and make a difference: “*They’re naturally passionate about it* [patient care] *for no personal reason, no personal gain, no positional gain whatsoever …*” (Hip fracture senior staff member 1). Healthcare assistants, ward domestics and assistants showed a strong sense of awareness of the importance of core patient-centred values in the caring process by talking about looking “*at the patients as if it’s my mum or my dad in that bed*” (Staff on Stroke pathway 2).

However, on both pathways, staff general attitudes towards patients were causing concern and compounding issues of poor communication and patient care:

“*We’d got lots of complaints about staff attitude, we’d got lots of complaints about patient care generally and I felt that if we engaged with the relatives more, if we involved them more that we would reduce the number of complaints*” (Stroke staff member 4).

In mediating these communication issues, staff hierarchies could play a further part in the communication difficulties in different ways.

One patient said: “*I feel more confident talking to a nurse about my symptoms than I do the doctor because it seems like the doctors are just … probably not their fault, they’re absolutely rushed off their feet but it’s very, boom, boom, boom, move on*” (Stroke ward patient 15). On both the stroke and hip fracture pathways there was also evidence of the importance of the role of domestics and ward assistants in communicating with patients and relatives in a supportive and less authoritative way:

“*The domestics, they go in, they just go into bay one and I’m not kidding you, they’ve had a massive laugh in there, all the patients get on, the families have joined it, they’re all having a bit of banter about what they’re going to have to drink, what they’re going to have to eat and the tea lady, oh she was amazing, but it bucks everybody up … the tea lady, the domestic, they would come and tell you, ‘oh she’s not very comfortable in the bed, can you go to her’ … so they are your eyes and ears as well*” (Senior stroke staff member 4).

“*For the dementia patients, we ask the family to give us information on how they were before and we can use that information then so we know the patient before she had the dementia and we can talk to her about her husband*” (Hip fracture health support worker 5).

At both sites, management talked about finding staff with the right values to promote patient-centred care and challenge paternalism through actively recruiting “*people with the values and skills the organisation wanted*”, “*changing the culture through staff leaving, and ongoing staff management processes*” and through “*supporting staff, by trying to make things better for them as well as for the patients*” (Senior staff member, Stroke pathway 6).

“*Whether it’s departmental culture or organisational culture, it’s rooted in the beliefs and values of the staff that actually work within that department, and it’s very difficult to change beliefs and values …, very difficult to change medical values when they’ve been entrenched in 300 years of training, but what you can do is to recruit people with the values that you believe are important, and that’s how you then change an organisation*” (Doctor, Hip fracture pathway 7).

However, social context, staff team and organisational systems also shaped the relationships that staff had with patients and relatives. Multidisciplinary teamwork was generally noted by most staff at both sites as important and supportive. This enabled them to tackle the complexity of patient care across pathways stretching from AE through to the ward and discharge, while juggling competing demands and shared resources. Key enablers in this process included promoting a strong team culture, shared values, interdisciplinary teamwork and ward rounds involving various healthcare staff (e.g. doctors, nurses, healthcare assistants, physiotherapists/occupational therapists, domestics, etc.) and support from colleagues. A senior practitioner on the hip fracture pathway thought that having a supportive team open to change was more important in getting her job done than the support she received from the wider organisational environment: “*We do bounce an awful lot off each other and support each other, and we are very close, because sometimes it’s a very difficult job*” (Senior hip fracture staff member 8).

On the stroke pathway, different staff spoke about being prepared to take on roles that went much wider than their job descriptions, because if they just stuck to their job descriptions, the “*ward wouldn’t function*”. Some senior staff saw themselves as patient advocates, in trying to challenge systems that disempowered patients: “*I shout at bed managers because that’s my job, I have to be a voice for my stroke patients. They don’t like me but I’m not here to please … if somebody is in the bed where a stroke patient should come, the bed manager will get a roasting from myself*” (Stroke staff member 9).

Structural barriers also impacted teamwork as staff described a lack of support to attend meetings and share concerns. Hierarchies of power and authority within teams could considerably impact teamwork relations: “*So this is what happens in a meeting, when you have a multidisciplinary meeting, the surgeon responsible actually has an interaction with a patient for probably the least amount of time from the other members of staff and yet would dominate 90% of the decision-making process or the influence, you know*” (Doctor, Hip fracture pathway 7).

This situation could particularly impact more junior members of staff: “*I suppose we have only limited power, however much we say. I suppose if it is not in the right tune, I suppose it just goes onto deaf ears*” (Stroke staff member 10).

On the hip fracture pathway an Advanced Nursing Practitioner described how a lack of continuity of care on the wards, aggravated by 12 h nursing shifts, made it much harder for “*teamwork and ownership*” to happen and to get the information needed because you “*haven’t got the same nurse looking after the same patient more than one or two days in a row*” (11). On the stroke pathway, more regular team meetings were suggested to improve communications between staff, but some staff feared this would just increase their workload. Staff at both sites talked about wanting to be valued more, rather than being told “*what’s wrong*”. A senior hip fracture staff member said: “*We’ve obviously got huge concerns for our staff because it’s a speciality you burn out in and  the pressures over the last couple of years have been immense*” (12). To try and alleviate this stress, the organisation provided the support of a wellbeing team, chaplain support, physiotherapy and massage services, and a drop-in clinic with a senior nurse to discuss any problems. However, several staff suggested that what was crucial to them was to address the structural and organisational issues impacting upon the provision of care that were often outside of their control, as described below.

### What systems/structures and processes enable/impede individuals and teams in improving patient experiences?

Prior to the King’s Fund PFCC research being conducted, staff highlighted the considerable improvement efforts that had been made at both case study sites to improve the quality of clinical care for patients. A large amount of work had been undertaken to streamline clinical pathways and improve services, using national quality indicators and guidelines to align with efficiency and productivity requirements. The King’s Fund PFCC work built upon these developments; however, despite the previous improvement work undertaken, staff at both sites still described many factors that disempowered them and which had a considerable impact in delivering good PFCC. These issues were related to wider structural and systemic factors. Staff frequently talked about feeling powerless or struggling to address these wider structural issues in managing patient care.

Staff shortages were a major issue, compounded by having to manage some very vulnerable patients on stroke and hip fracture wards. On one hip fracture ward, 10 patients from a 30-bed ward had dementia and 6 staff were absent, including 2 on maternity leave. Staff at this site described considerable shortages with therapists who were operating 3 times over capacity. Managing the pressure of patient flow through the hospital, exacerbated by a lack of theatre capacity and shortage of beds on wards or patients ready for discharge still occupying beds, was also a major problem. On the hip fracture pathway staff felt it was not possible for them to change the lack of beds or flow problems in AE, as this issue was part of a wider system problem: “*What we can’t do is influence the patients going out, from my perspective in the ED I can’t get people home faster*”. The point was also made that the way health service funding worked in Wales at the time of the research may have actually exacerbated these problems as budgets stayed the same no matter how many patients came through the door, “*so there’s no impetus to do more work*”. Similarly, with respect to managing discharge, staff found themselves negotiating systems in caring for patients that they had little control over, but which was hugely time consuming:

“*You are dependent, as an acute unit on making sure that the family makes those arrangements, that social services make those arrangements, and that there’s a space available for wherever they’re going to go* (e.g. community hospitals)” (Hip fracture staff member 13).

At the time of conducting the research, one staff focus group at the Wales site found that 13 out of 26 beds on the trauma ward were being occupied by patients who were ready to go home. Patients and staff also talked about the importance of the ward environment and patients having access to things such as a day room, comfortable chairs, TV’s they did not have to pay for and facilities like access to a wheelchair to get to the hospital shop, newspapers, radio, a chiropodist, etc., which could make a big difference to a patient’s recovery, particularly for elderly patients. These were facilities that staff said in the current cost climate they often had to fundraise for. Broader structural issues also concerned the quality of the food service, noise, and ward layout leading to patients having to be moved about to ensure single-sex wards, subsequently impacting on continuity of care.

The limited resources of the hospital system particularly came to the fore when discharging patients on the stroke pathway. Patients spoke of being discharged quickly without sufficient information and, sometimes, without enough resources to be able to cope in the community. The relative of a stroke patient (17) contrasted the Trust’s “*excellent*” mission statement on discharge, with what happened to his wife in practice “*they broke every facet of the mission statement*”. Conversely, in the hospital itself, there was significant pressure for speedy discharges as reducing length of stay and ensuring that there were enough beds for new stroke patients was vital. With the hip fracture pathway, whilst most patients and relatives seemed to be mostly content with the discharge process itself, the main problems occurring seemed to be with long waits to be discharged from hospital, finding rehabilitation beds in the local areas and the complex communication and processes involved in these siuations:

“*We had a patient here, she was medically fit, for 41 days. But what happens is more often than not – which is fair enough – is that patients live in their own home, you've got the family that’s caring for them, they obviously work, it’s very difficult, so then they fall. They can’t go to rehab because they’ve got dementia, so the next step then would be a nursing home, probably, but there is another process then to go through and it’s a long process … if they’re self-funding I suppose it isn’t that bad but if they need to be funded you’ve got to have meetings, a meeting with the family and the social worker, the physio, the nurses. You’ve got all this information there you’ve got to gather to send to the social worker*” (Hip Fracture, senior staff member 14).

At both sites, there was an acknowledgement that staff could try to mitigate the effects on patients of systemic pressures by communicating and explaining problems and issues with patients. However, time and space for this communication was increasingly squeezed with staff who were already feeling disempowered by the organisational issues described above.

### What outcome measures enable agents to understand if changes have impacted patient and family experiences of care?

The outcome measures that were being used by the two case study sites to evaluate experiences of care were found to influence and set particular limits, trajectories and priorities for improving patient care. Specific measures included patient experience and nationally introduced clinical audits and outcome measures. On the stroke pathway, this included the Safe Implementation of Treatments in Stroke audit for any patient that has undergone thrombolysis and the Sentinel Stroke National Audit Programme for any patient that had been on the stroke pathway. These audits included data such as how many patients are scanned within 1 hour, how many patients are admitted to a stroke unit within 4 hours, how many stay on the specialist stroke unit and data on discharge processes. Mortality figures and length of stay were also key measures that were carefully monitored in addition to the number of and types of complaints. On the hip fracture pathway, the main reporting indices were 30-day mortality, acute length of stay and the average time to surgery with the percentage of patients receiving surgery within 24 hours.

The King’s Fund PFCC improvement methodology required teams to identify specific project aims and a small number of locally devised before and after measures, as part of a Plan, Do, Study, Act (PDSA) cycle, to enable them to monitor their own progress on locally devised improvement projects. However, in practice, teams on both pathways defaulted to the use of the national clinical outcome measures that were already being used on the pathways, rather than develop specific PFCC improvement measures. It was therefore not possible to measure the specific impact of the PFCC improvement work. Both sites also used their own organisational patient experience surveys to evaluate patient experience; however, problems were identified with how this data was collected and managed. On the main stroke ward, the ward policy was to give questionnaires to all patients upon discharge with the aim of capturing feedback from 80% to 90% of patients. In practice, staff tended to complete the forms with patients on the ward because they were not well enough to do this on their own or, because of short staffing, the ward administrator randomly chose patients who were capable of participating. This meant that only about 15% of patient feedback was captured and the whole process of completion raised questions about the independence of the feedback, the lack of capacity of some patients to fill in the forms and support for staff in completing the process with vulnerable patients.

In the hip fracture services, similar issues also arose with the way in which patient experience measures were collected and used. The main hip fracture ward was required to hand out patient experience forms to all in-patients on the ward once a month and had to collect at least 16 (it was formally 30), out of a possible 90 forms. The data was then collated and displayed on the ward notice board, although not discussed in staff meetings. Whilst the ward sister felt that she had been able to pick up on key issues and make lots of changes as a result of this feedback, another member of staff noted that she “*did not think staff generally looked at the charts on patient experience*”. She thought it would be good for the feedback to be discussed at morning handover meetings, which would enable healthcare assistants and nurses to participate. The second trauma ward on the hip fracture pathway stated that they did not collect patient feedback. A staff member interviewed said: “*I know we have got the patient surveys although I have to hold my hands up and say I don’t know where they are and I’ve never given one out*”.

More generally, while 33 patients and 8 relatives participated in the research study (12 being very happy with their care and 21 describing a mixture of both good and poor care), none of the patients interviewed said they had been asked to complete patient feedback and experience forms as part of the process of gathering patient feedback as described above. Despite the concerns raised by interviewees, only one person had made a formal complaint, which she felt had been dealt with very negatively, “*I’ve said I haven’t wanted to complain in a negative way, I’ve wanted to raise concerns so that things can be made better*”. No other complaints data appeared to exist for these pathways.

These results illustrate the difficulties that the case organisations had in collecting, collating and acting on patient experience evidence and measures. The way this was being conducted would not have supported staff in evaluating patient experience in any comprehensive way. Clinical effectiveness measures still dominated the understanding of improvement work and these measures became the signposts for patient experiences; this tended to reinforce the dominance of clinical effectiveness in comparison to patient experience, when understanding and improving healthcare quality. This situation was compounded by the fact that no patients nor the public were involved in any of the hospital-based PFCC work at either of the sites.

Despite this situation, both sites described the PFCC work undertaken as supporting them to think more broadly beyond the clinical indicators being used. On the stroke pathway, an early PFCC study session enabled staff to think about the links between poor staff experience and its impact on patient experience, with further work being done to improve staff experiences. On the hip fracture pathway, staff described how new perspectives and patient-centred values emerged from the PFCC project shadowing and care process mapping. This enabled clinical staff to gain a much wider perspective on the whole service pathway from patient perspectives; this was information that the King’s Fund PFCC work expected staff to act on locally.

### Patient and public involvement

A key weakness in the PFCC work was that it did not include the involvement of any patients and the public in the processes of improvement. Some staff noted that, whilst they wanted to involve patients to a greater extent, it was difficult to do this in relation to the resources, skills and time available. The study findings illustrate that, in practice, there were clearly a huge number of context issues with staff having the right infrastructure to support PFCC and involvement. One way that greater partnership working was enabled between researchers, lay members and staff in the research was to draw upon a PAR approach. This methodology allowed us to address some of the power imbalances in conducting the research through RAGs for each pathway (described in the Methodology section).

Whilst this more collaborative process greatly enriched the research design, it clearly provided a starting and not an endpoint for developing more equal collaboration. Future learning in building on this process would be to start earlier to gain the involvement of lay perspectives into the process and to ensure that methods supported the inclusion of both patients with personal experiences as well as those working for voluntary organisations and the input of a wider diversity of patients. It is also important to address hierarchies in partnership working and to ensure more input is gained from junior staff as well as senior members and from BAME and other under-represented groups.

## Discussion

These findings illustrate how organisational structures and the power dynamics within them act as contextual constraints [37] that limit improvements in patient experiences at every level of the system. Despite this, there is little mention of how to address and mitigate these power inequities in improvement methods such as PFCC and the national policy frameworks guiding patient experience. By focussing on power relations, and the broader structural contexts within which staff work, this article has illustrated how power inequities can be embedded at all system levels (as illustrated in Table 3 below).

**Table 3:** Key power dynamics and considerations for improving patient experiences using a whole-systems approach

**Power relations (individual agency) (What can individual staff do to improve patient experience?)**	**Micro level findings**
• Shift from paternalism to patient empowerment • Share power in decision-making • Move from hierarchical teamwork to a more collaborative approach	• Patient-centred values and approach (as opposed to paternalistic care) as the foundation for individual care • Shared communication and involvement in decision-making • The provision of relevant information to patients and relatives • Interdisciplinary teamwork with shared values
**Power in organisational systems (what can organisational managers and teams do to improve patient experience?)**	**Meso level findings**
• Empower staff through resources/tools/knowledge that support improvement • Empower staff through time to care • Enabling interdisciplinary/non-discriminatory ways of working • Organisational support that empowers, rather than disempower staff/patients • Partnership working and co-production	• The development and support of relevant improvement initiatives (connected to quality, safety and clinical effectiveness) to improve care • Staff time, support, training and development to provide patient-centred care and improvement and the involvement of diverse groups of patients and relatives • Organisational and managerial support for interdisciplinary/non-discriminatory teamwork • Organisational context, structures and infrastructure in supporting the care process (e.g. resources, physical environment, patient turnover, systems for clinical care and discharge) • Developing systems to support partnership models of diverse patient and public involvement
**Structural power at the external national/policy level (what influences do national policies have on organisations’ ability to improve patient experience?)**	**Macro level findings**
• Financial/resource constraints • Policy/legislation needs to drive local staff/patient empowerment • Incentives for organisational systems	• Tighter levels of finance/resources can provide contextual constraints for transactional and relational aspects of care • Policy/legislation can promote or inhibit good practice but needs to be regularly reviewed to ensure it is meeting intended goals for patient experience, e.g. patient experience surveys not being collected because they are mandatory rather than usefully capturing patient feedback • National targets and measures can provide an important benchmarking system that encourages staff within organisations to act to improve aspects of services • National measures seemed to have a stronger motivational force than internal measures related to improvement projects

This article suggests that more attention needs to be paid to the structural contexts within which staff act [35], and how these may limit the improvements that they can make to patient experiences. These issues need to be addressed at multiple levels to empower both patients and healthcare staff.

At the level of direct care, this study has highlighted various examples of power inequities and paternalistic care by doctors and nurses with patients and relatives. This manifested in rude and uncaring attitudes and behaviours, poor communication that did not allow patients and relatives to feel listened too, to discuss their concerns and to be given adequate information, and time to discuss their clinical condition and treatment. In contrast, there were also various examples of good person-centred care which highlighted behaviour that was kind, caring, attentive and compassionate, built on a patient-centred approach.

A number of staff cited time and system pressures as constraining the degree to which they could communicate effectively with patients. Other research shows how shorter lengths of stay mean less time and space for good communications between staff and patients [56]. This was illustrated in our findings, where speedy stroke discharge processes gave little time for discussion with patients. Length of stay targets were stronger cultural drivers than patient voices. At times, staff did not have the time or resources to be able to provide PFCC because they were working to the system demands.

In improving patient experiences, staff could be hampered by wider contextual constraints that limited their actions due to multiple competing demands within a resource-constrained system. Local issues raised within the two case studies also mirrored broader healthcare system problems that currently affect and constrain NHS Trusts nationally. This includes national AE pressures [57, 58] and within hip fracture care, once patients are medically fit for discharge, their hospital length of stay is determined by factors such as local care home supply, which are outside of a hospital’s control [59].

These types of national issues were not easily remedied through small PFCC interdisciplinary working groups. These groups did not have the power and resources to tackle problems that were symptomatic of much larger organisational or national constraints or issues. However, despite organisational pressures, some staff explained how good communications could reduce the adverse impact of system priorities on patients. Multidisciplinary teamwork was also noted by staff at both sites as enabling them to tackle the complexity of patient care across pathways stretching from AE through to the ward and discharge, while juggling competing demands and shared resources.

For patients to be active partners in their care, they need power and influence within decision-making. Patient empowerment (focused on the role of people who receive health or care services and their carers’ and families), is seen as a solution to many of the most pressing problems facing modern healthcare. Yet, our findings illustrate how patient empowerment has not always translated into patients' everyday experiences [60]. Around half of English patients in hospitals say they are not involved in decisions about their care as much as they would like — a figure that has shown very little improvement over the last 15 years [61]. Our results suggest that both structural capacity problems and individual attitudes and values need to be addressed to tackle these issues.

The findings from the All Party Parliamentary Group on Global Health in 2014 reflect this thinking in arguing that “*the biggest challenge for the NHS is to go beyond isolated initiatives to a whole-system effort*” ([60], p. 3), and that the Government is right to set NHS England the challenge of becoming “*dramatically better*” at involving patients and their carers [60, 62]. This study shows that patient experiences can be affected by the whole hospital and policy system, and therefore that improvement efforts need to take a whole system approach to improvement (as outlined from our findings in Table 3). In this way, our findings concur with Dixon-Woods and Martin [63], that small-scale improvement projects may lack the necessary power and resources to make changes to improve patient experience and quality.

It is only when there is sufficient individual and organisational capacity that patient feedback may be acted on to improve patient experiences [64]. Viewing care from the patient perspective was seen as a real ‘eye-opener’ at one site where mapping ideal patient pathways and shadowing was considered a helpful and supportive tool to improve services. However, it was not clear how much data from shadowing was used to make changes in practice. Patient experience surveys were often carried out inconsistently or lacked independence and were not shared with all staff. Patient experience data and the quantitative clinical measures did not always provide staff with the necessary in-depth knowledge that was needed to identify how to improve patient experiences. On both the hip fracture and stroke pathways, organisational and national contextual constraints ultimately appeared to act as the most important barrier to implementing broader improvements to patient experience. However, for service improvements to be sustained, they need front line clinical staff engagement to implement change and develop practice [65]. Improvement activities need to be focused at all levels within a healthcare system.

### Implications for developing patient experience

These findings suggest that improving patient experience requires a more critical whole-systems approach. This approach needs to address the systemic nature and complexity of organisational factors and power relations that can empower and disempower patients and their families as well as healthcare staff in improvement processes.

Healthcare contexts are non-linear, emergent and dynamic [66]; patient experience frameworks need to develop to account for this. Systems approaches can help us to understand the complexity of social processes, enabling us to study different system components and their relationships within a wider environment [67]. A systems approach should be further utilised within patient experience and involvement thinking and practice to take greater account of context [68].

To further develop patient experiences, we need to understand how power operates at both a macro level, where national and institutional policies and resources may constrain or enable action, and at a micro level, where staff may be able or unable to change services within their situated contexts. In this latter situation, there is a need to create empowering social change, to support health professionals and in turn to create more empowering and equitable relationships with patients. The current Patient Experience Framework [17], for example, needs to provide more examples of the ways in which the Public Sector Equality Duty can be implemented in practice to support the development of more diverse and inclusive relationships and partnerships in healthcare organisations.

Ultimately, the strength of the PFCC method lies in its focus on understanding care experiences through patients’ eyes, building on staff’s empathy and connectedness, and to encourage interdisciplinary working to improve patient-centred care. However, a weakness of the approach is its ability to take into account power differentials between different groups of staff, power inequities between diverse patient groups and staff, and how patients and the public can be actively empowered to be part of the improvement agenda in practice.

A key recommendation given the lack of PPI in improvement work in the study, is that the PFCC method is further developed to engage both patients and the public and staff collaboratively in improving services. Experience-based co-design and coproduction approaches provide examples of how to engage patients and staff in improving services [69, 70]. Coproduction approaches are increasingly seen as a way to address power inequities in collaborating with patients and the public [71]. However, further work is needed to ensure that imbalances of power between healthcare staff, patients, public and organisations as well as issues of equality and diversity are addressed within these collaborative approaches [26, 72]. The testing of approaches that can involve a more diverse representation of patients, the public and healthcare staff in line with the protected characteristics in the Equality Act 2010 [73] (e.g. age, disability, gender, race, religion or belief, sexual orientation), to see what works best, is a considerable gap that needs to be addressed in current improvement practice. These methods for more diverse and inclusive partnership are long overdue and should be developed and built into the structures of all healthcare organisations in the near future.

### Strengths and limitations

The strengths of this study were that it was able to use multiple methods to study improvements in PFCC at the micro, meso and macro level, across two different case study sites in England and Wales. The use of qualitative research methods, such as drawing on patient stories, interviews and focus groups and observations as well as co-produced and collaborative work with patients and the public, also provided much greater insight than just relying upon quantitative data to look at these issues. The research study also used a participatory approach to empower a wider group of stakeholders to gather information from different perspectives. PAR can provide a practical approach for addressing power inequities in developing collaborative relationships in healthcare improvement. Using these mixed methods, the study was able to highlight the limitations of current patient experience and PFCC methods and the need to adopt broader approaches that address power inequities in involving patients, relatives, carers and staff in improvement processes.

The study was limited in only looking at 2 case studies out of the 11 sites that were part of the King’s Fund PFCC project; they may therefore have been untypical of the other sites involved. The collaborative processes with staff and patients also tended to involve White staff and patients and therefore wider strategies need to be developed for involving groups from across the protected characteristics.

## Conclusion

Patient experiences are often understood and measured using positivist linear approaches. However, healthcare experiences are often affected by power relations at the individual/team, system/process and national levels. Healthcare staff and small-scale service improvement projects (that can completely exclude patient and public involvement), may not have the necessary power or resources to tackle key aspects of patient experience because they are affected and impacted by wider organisational systems and national forces. This article has illustrated how organisational and policy contexts affect the implementation of patient experience initiatives. Issues of power and context are not accounted for sufficiently in current policy and models of patient experience, involvement and PFCC frameworks.

## Data Availability

The datasets generated during the current study are not publicly available due to the confidential nature of the participant interviews and the data containing information that could compromise research participant privacy/consent. Consent to share anonymised interview transcripts was not asked for at the time of the research.

## Appendix 1

### Background on the stroke and hip fracture pathways

#### Hip fracture pathway

The hospital where the hip fracture pathway is based provides a comprehensive orthopaedic trauma service covering two acute sites. The main site treats up to 400 patients with a hip fracture per annum. The care pathway key performance indicators are reported to the National Hip Fracture Database. Hip fractures are a common consequence of falls in the elderly population,, often affecting those above the age of 60 with a median age of 75. Hip fractures in the elderly are one of the greatest challenges facing orthopaedics, with a rising incidence of 8–10% per annum due to an increase in the aging population and medical comorbidities. The majority of patients require surgical treatment but, despite this, around 10% of patients will not survive more than 30 days following the injury.

#### Stroke pathway

The stroke pathway is made up of a combined acute stroke unit (hyper acute beds and acute beds) and a specialist stroke rehabilitation unit. There is a daily rapid-access TIA (transient ischaemic attack) clinic, closely linked with vascular surgery with 24/7 access to CT, CT angiography, perfusion and diffusion imaging, and MRI. The Centre is one of the most active in the region. There are two main causes of strokes — ischaemic, where the blood supply is stopped because of a blood clot, accounting for 85% of all cases, and haemorrhagic, where a weakened blood vessel supplying the brain bursts. The stroke pathway supported both types. Stroke can affect people of all ages, including children. Many people with ischemic strokes are older (60 or more years old) and the risk of stroke increases with age.

## Appendix 2

### Coding and analytic framework

1. Macro – wider structures impacting patient experience:
  - Policy
  - Mandated practice (including financial)
2. Meso – organisational context:
  - Physical
  - Social systems/methods
  - Power and authority structures and roles
  - Resources
3. Culture as a part of meso organisational context, including:
  - Organisational values
  - Values promoting a learning environment
  - Values of diversity/inclusivity/empowerment with respect to staff/patients
4. Specific clinical contexts within the hip fracture/stroke pathway:
  - Ambulance
  - Accident and emergency
  - Diagnosis
  - Specific treatments according to pathway (pain relief, X-ray and theatre for hip fracture and specific stroke treatments related to haemorrhagic stroke and transient ischaemic attack)
  - Ward (cleanliness, food)
  - Physiotherapy and occupational therapy
  - Discharge process
  - Post-discharge experiences
5. Mechanisms/processes impacting patient experience:
  - Individual/team values/beliefs/behaviours
  - Receptiveness to change
  - Power and decision-making
  - Communications
  - Leadership at organisational/team/individual level
  - Effective teamwork/relations (e.g. clarity of aim/purpose with team’s work)
  - Use/effectiveness of facilitators as catalysts for change
6. Outcomes impacting patient experience
  - Measurable change/improvement
  - Behavioural/values change (individual/team change)
  - Clinical processes
  - Teamwork/partnerships

## Abbreviations

AE: accident and emergency
BAME: Black, Asian and minority ethnic
PAR: participatory action research
PFCC: patient- and family-centred care
PPI: patient and public involvement
